# Supplementary figures and images for: OsbHLH067, OsbHLH068, and OsbHLH069 redundantly regulate inflorescence axillary meristem formation in rice
Source: PLoS Genet. 2023 Apr 13;19(4):e1010698. doi: 10.1371/journal.pgen.1010698 (PMC10128955; doi:10.1371/journal.pgen.1010698)

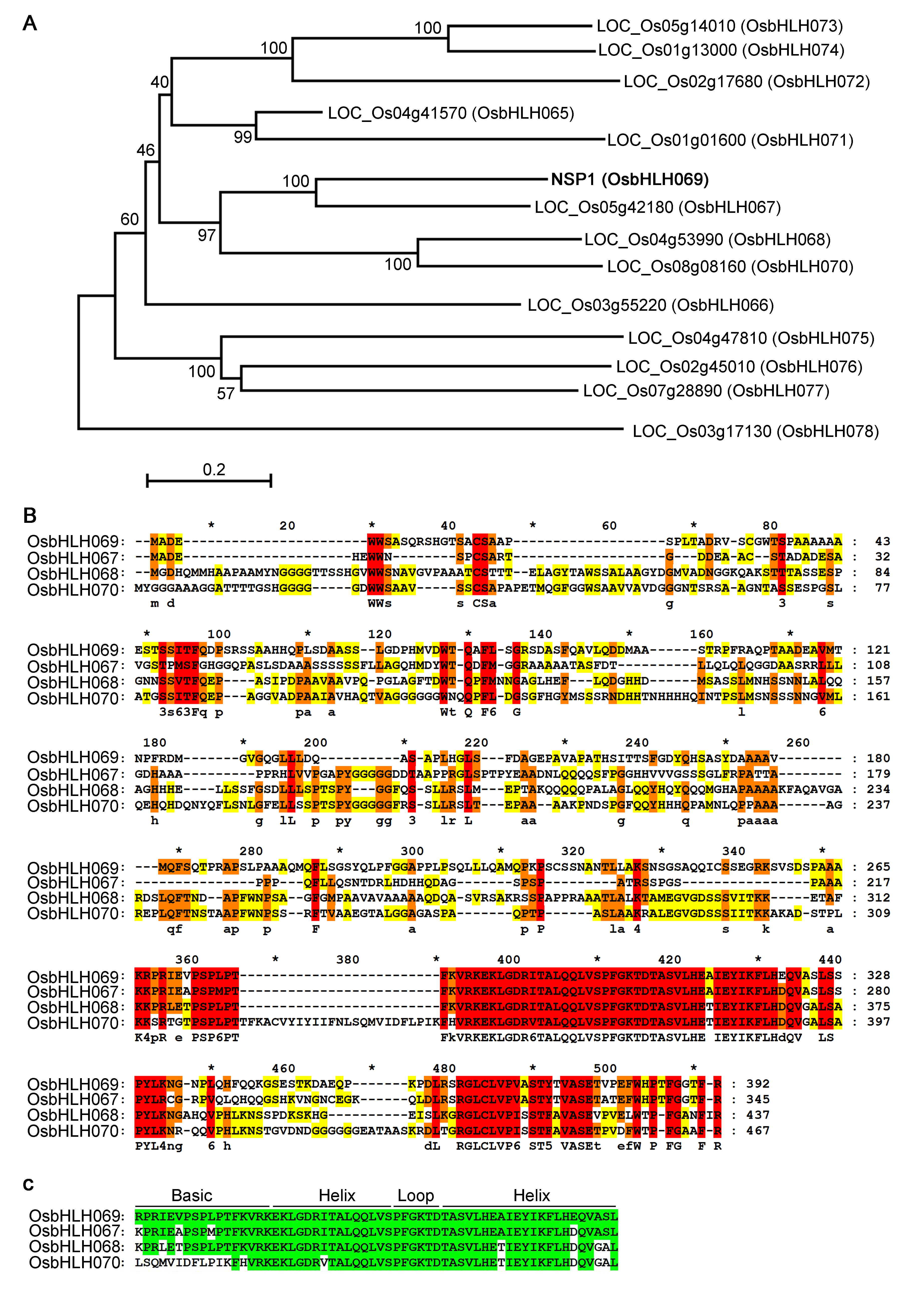

Supplement: S1 Fig — (A) Phylogenetic analysis of putative OsbHLH069 homologs in rice using MEGA5.1 with neighbor-joining method and the following parameters: Poisson correction, pairwise deletion, and bootstrap (1,000 replicates; random seed). (B) Alignment of OsbHLH067, OsbHLH068, OsbHLH069, and OsbHLH070. Alignment was conducted with ClustalW from a MEGA5.1 program and then mapped using GeneDOC software. Red, orange, and yellow shading represent residues conserved in 100%, 80%, and 60% of the sequences, respectively. (C) Comparison of the bHLH domain of OsbHLH067, OsbHLH068, OsbHLH069, and OsbHLH070. (TIF) [file pgen.1010698.s001.tif]

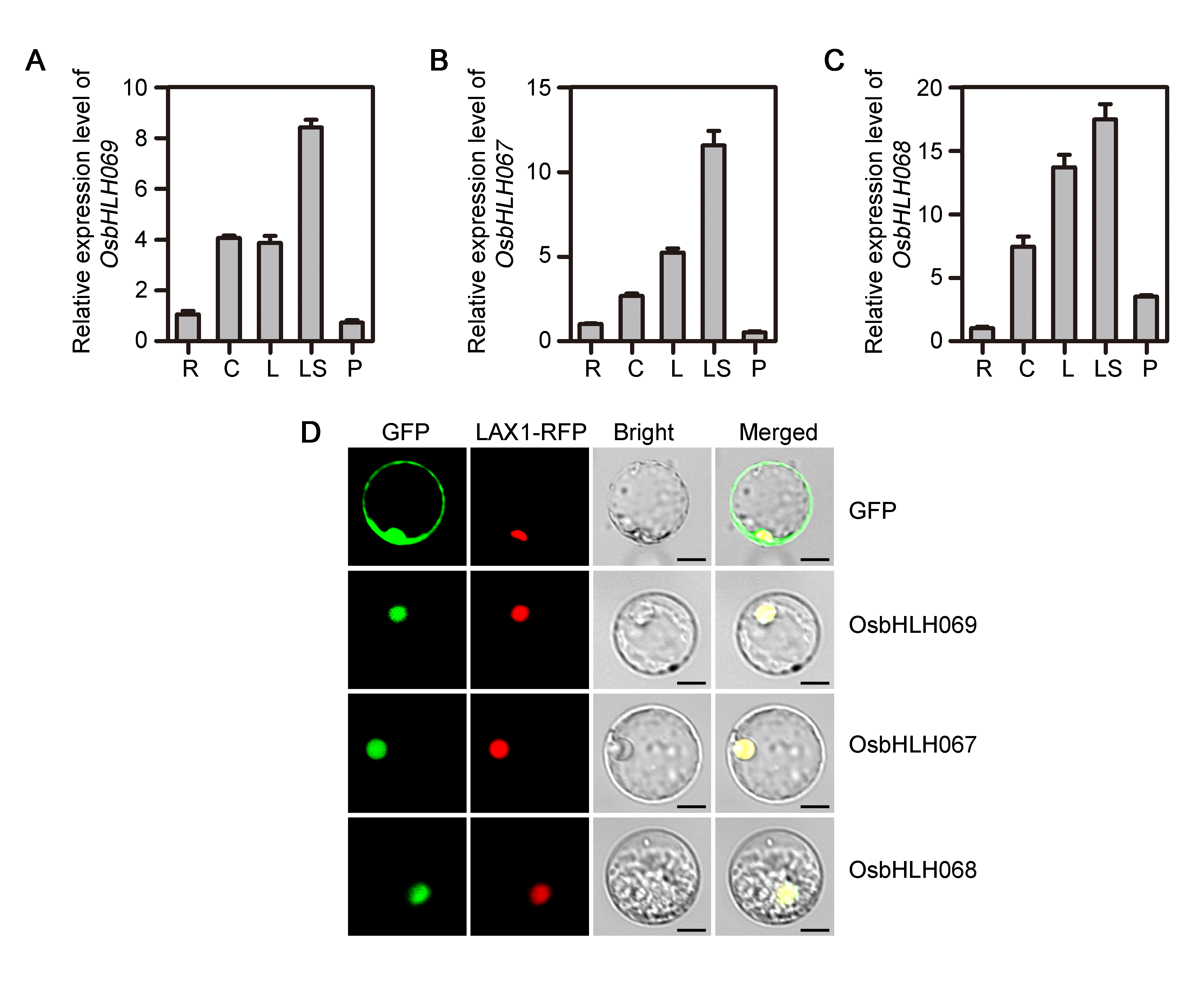

Supplement: S2 Fig — (A) to (C) Expression levels of OsbHLH069 (A), OsbHLH067 (B), and OsbHLH068 (C) in various organs, including R (root), C (culm), L (leaf), LS (leaf sheath), and P (panicle < 5 mm). Rice UBQ gene acted as a control. Values represent means ± SEM from nine replicates. (D) Subcellular localization of OsbHLH069, OsbHLH067, and OsbHLH068 in rice protoplasts. The LAX1-RFP vector was used as a nuclear marker. The 35S-GFP vector served as a control. Bars = 10 μm. (TIF) [file pgen.1010698.s002.tif]

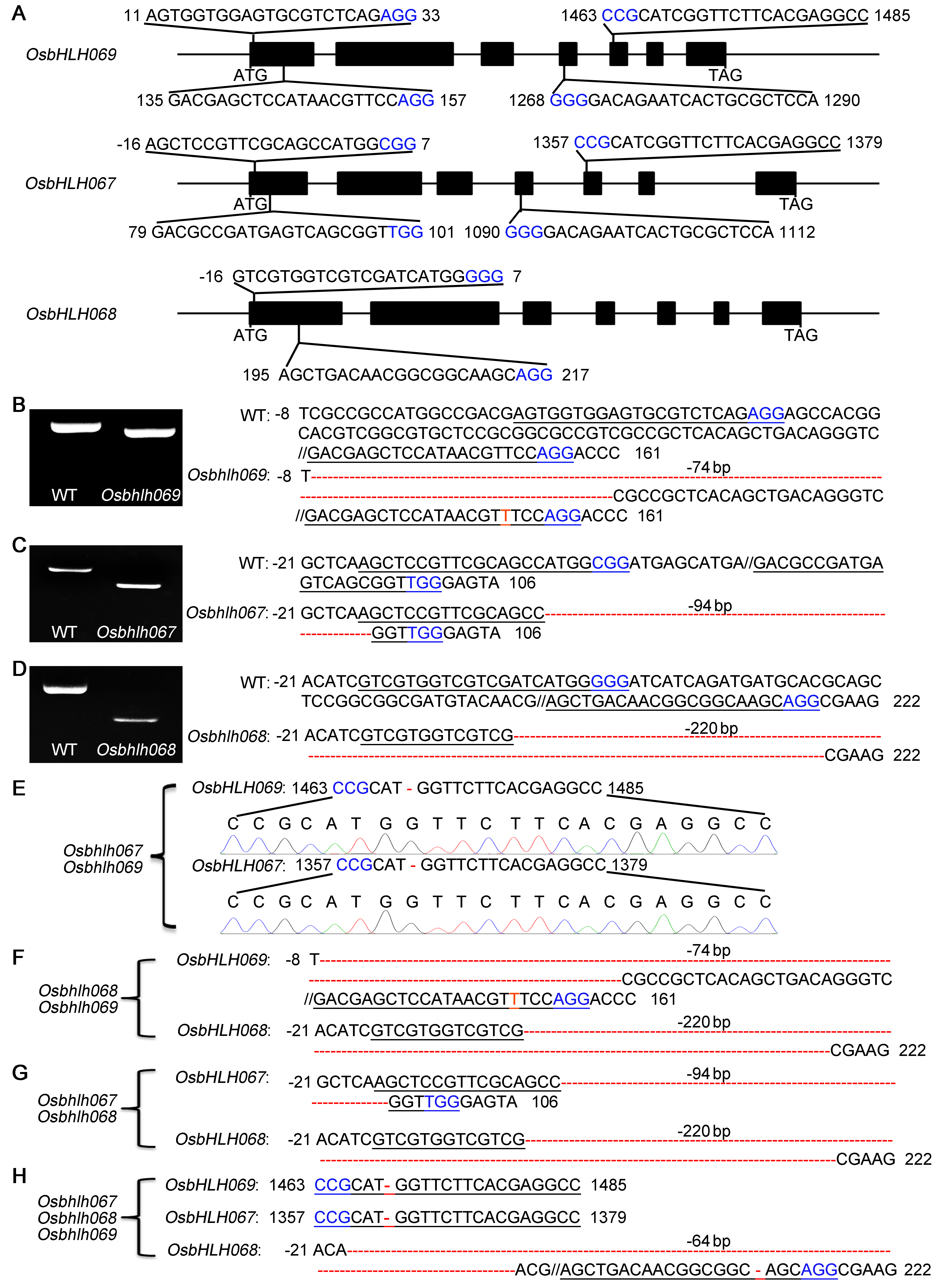

Supplement: S3 Fig — (A) Schematic diagram of sgRNA sites of OsbHLH069, OsbHLH067, and OsbHLH068 by CRISPR/Cas9 system, respectively. Boxes denote exons, and lines between the boxes indicate introns. (B) to (H) Analysis of the mutation sites in single mutants Osbhlh069 (B), Osbhlh067 (C), Osbhlh068 (D), double mutants of Osbhlh067 Osbhlh069 (E), Osbhlh068 Osbhlh069 (F), Osbhlh067 Osbhlh068 (G), and triple mutant of Osbhlh067 Osbhlh068 Osbhlh069 (H). The red ellipsis represents the missing base; the double slash represents the omitted base; the bases underlined correspond to the target sequences; the bases in blue represent PAM. (TIF) [file pgen.1010698.s003.tif]

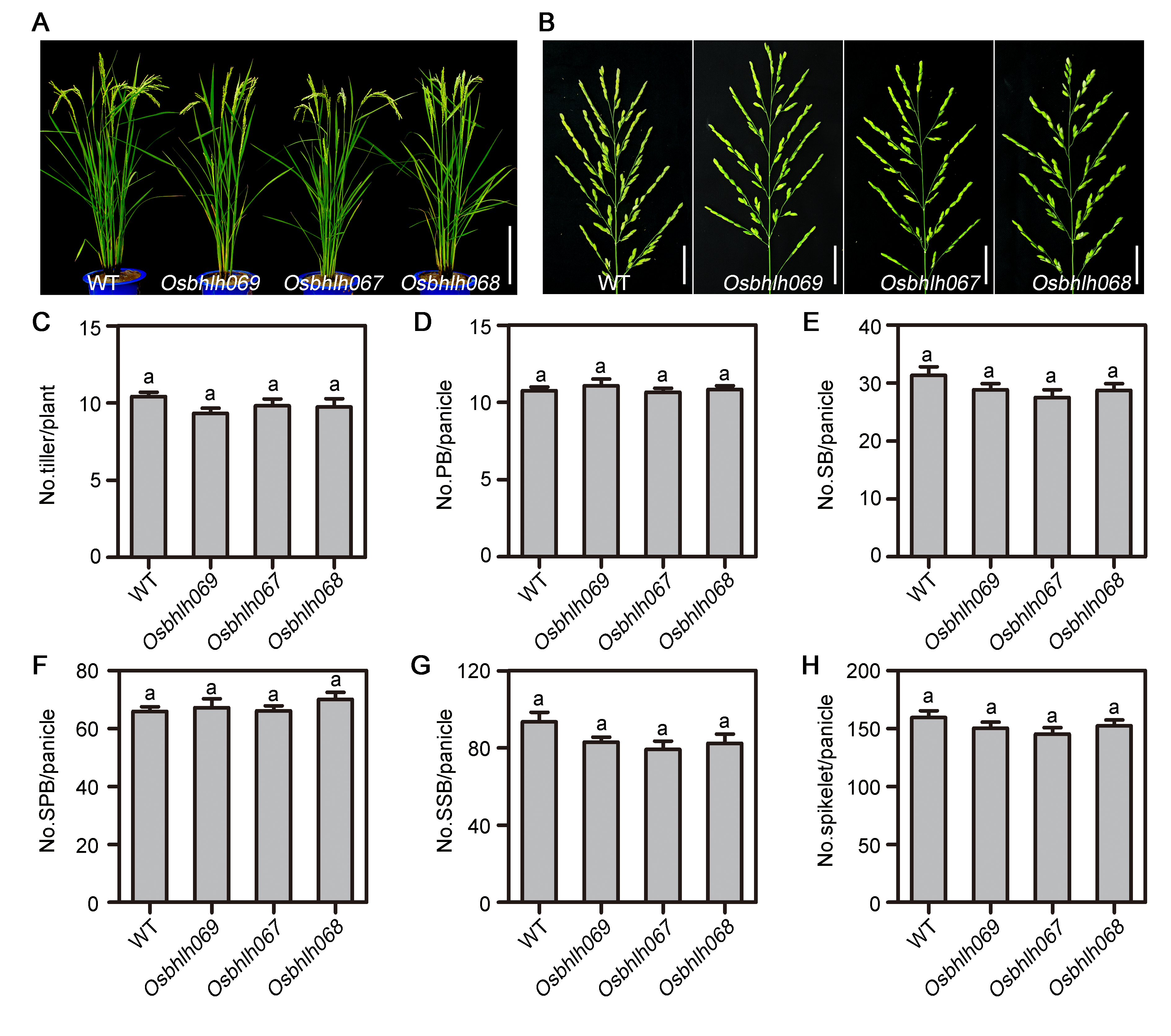

Supplement: S4 Fig — (A) and (B) Comparison of the gross plant (A) and panicle (B) among WT, Osbhlh067, Osbhlh068, and Osbhlh069 during reproductive growth. Bars in (A) and (B) = 20 cm and 4 cm, respectively. (C) to (H) Quantification of the number of tillers (C), PBs (D), SBs (E), SPBs (F), SSBs (G), and total spikelets (H) among WT, Osbhlh067, Osbhlh068, and Osbhlh069. Values in (C) to (H) are shown as means ± SEM from 12 replicates. Different letters denote significant differences ranked by the Dunnett’s test (one-way analysis of variance, P < 0.05). (TIF) [file pgen.1010698.s004.tif]

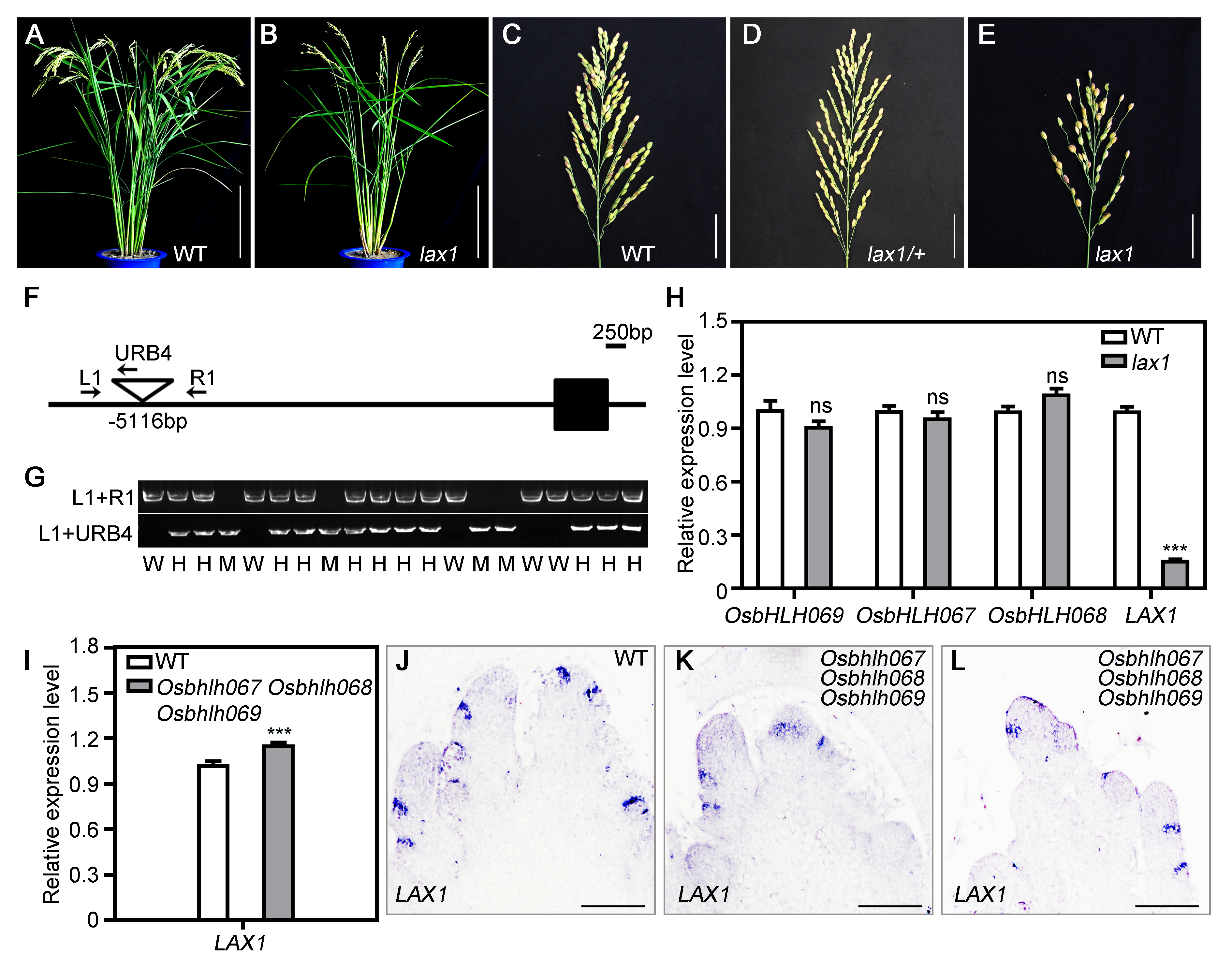

Supplement: S5 Fig — (A) and (B) Gross morphology of WT (A) and lax1 (B) during reproductive growth. Bars = 20 cm. (C) to (E) Panicle morphology of WT (C), lax1/+ (D), and lax1 (E). Bars = 4 cm. (F) The T-DNA insertion site in lax1. Box represents the LAX1 genome, and the triangle indicates T-DNA. The primers L1, R1, and URB4 used for genotype analysis are marked with arrows. (G) The co-segregation analysis of lax1. W, H, and M indicate WT, heterozygous, and homozygous for T-DNA insertion, respectively. (H) Expression analysis of OsbHLH069, OsbHLH067, OsbHLH068, and LAX1 in the young panicles (< 2 mm) of lax1. (I) Quantitative RT-PCR analysis of LAX1 expression in the young panicles (< 2 mm) of WT and triple mutant Osbhlh067 Osbhlh068 Osbhlh069. (J) to (L) In situ hybridization with a LAX1 probe on WT (J) and Osbhlh067 Osbhlh068 Osbhlh069 triple mutant (K, L) inflorescences at the differentiation stage of secondary branch meristem. Bars = 100 μm. The rice UBQ gene was used to normalize gene expression. The values shown in (H) and (I) are means ± SEM from nine replicates. Significant difference (two-tailed Student’s t-test, ***P < 0.001); ns indicates not significant (two-tailed Student’s t-test; P > 0.05). (TIF) [file pgen.1010698.s005.tif]

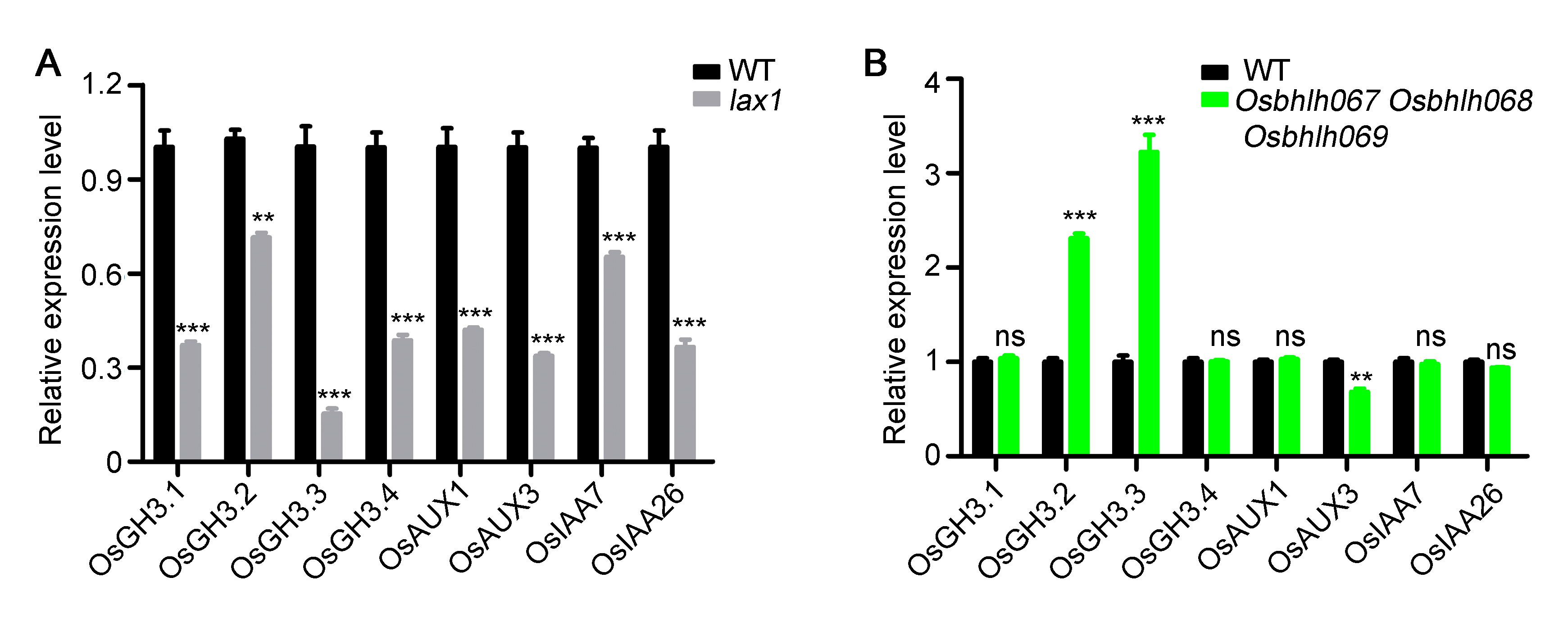

Supplement: S6 Fig — Quantitative RT-PCR analysis of eight auxin-related genes in the young panicles (< 2 mm) of lax1 (A) and Osbhlh067 Osbhlh068 Osbhlh069 triple mutants (B). Data were normalized to the rice UBQ gene, and values represent means ± SEM from three replicates. Significant difference (two-tailed Student’s t-test, **P < 0.01, ***P < 0.001); ns indicates not significant (two-tailed Student’s t-test; P > 0.05). (TIF) [file pgen.1010698.s006.tif]
