# Supplementary material for: OsbHLH067, OsbHLH068, and OsbHLH069 redundantly regulate inflorescence axillary meristem formation in rice
Source: PLoS Genet. 2023 Apr 13;19(4):e1010698. doi: 10.1371/journal.pgen.1010698 (PMC10128955; doi:10.1371/journal.pgen.1010698)
Supplement: Attachment — Submitted filename: Response to the reviewers.pdf [file pgen.1010698.s013.pdf]

Reviewer #1: General remarks:

1. Overall the language has to be improved. I see the problem not being native speaker (this applies to me too). Still somebody should try to correct the language.

Response:

Thanks for your comments.

Yes, we have improved the language with the assistance a native speaker.

2. The manuscript contains to many inconsistencies and errors. Material and methods are incomplete etc. I mention some of the problems at the end of the review, but not all. It is not the job of the reviewer to indicate all errors.

Response:

We checked the manuscript and corrected the relevant errors. We have added the methods pull-down assay, RT-PCR, and histological sectioning in the Material and methods.

Summary of the paper:

The authors provide valuable information on the following points. Most of the information provided may be helpful for other researchers in this field:

- The authors describe a novel rice mutant, nsp1-D, with reduced inflorescence branching. The mutant phenotype is semi-dominant.
- They give a detailed description of lateral meristem defects in heterozygous and homozygous mutant plants.
- This mutation is caused by the insertion of a T-DNA upstream of rice bHLH069 gene, causing over-expression of the gene. Insertion of the T-DNA co-segregated with the phenotypes and overexpressing bHLH069 by T-DNA-constructs led to reduction of inflorescence branching in varying amounts.
- bHLH069 is a member of a small group of related genes, including bHLH067 and bHLH68. qRT-PCR indicated, that the three genes are widely expressed, but in situ hybridization demonstrated preferential expression in shoot, inflorescence and floral meristems. Expression patterns differ from the expression pattern of the branching

regulator LAX1.

- Nuclear localization has been demonstrated for GFP fusion of all three bHLH proteins.
- Loss of function (LOF) alleles have been obtained for the three genes by CRISPR/Cas9 technology.
- Single LOF lines did not display phenotypic alterations. Similarly, double mutants had no or very weak defects in branching.
- Triple mutant plants displayed reduced height, as well as reduced shoot and inflorescence branching. Reduced inflorescence branching is linked to reduced expression of the meristem marker gene OSH1.
- The authors identify a new LAX1 loss of function allele in the isogenic background. The phenotype of this mutant is comparable to the phenotypes described for lax1-mutants described earlier.
- Combining lax-1 with nsp-1D resulted in enhanced lateral meristem defects. Heterozygous nsp1-D in lax-1 mutant background and heterozygous lax-1 in homozygous nsp1-D mutant background enhanced the phenotypic defects of the respective mutants. (statistical analysis seems to assume normal distribution of data – but at least for F,G,H, and I data are shifted towards).

Response:

Heterozygous *nsp1-D* shows a weak sparse inflorescence with reduced grain setting, and homozygous *nsp1-D* shows a serious sparse panicle, which is similar with *lax1*. The heterozygous *lax1* (*lax1*/+) performs a normal panicle (S5D, Fig). Thus, in the homozygous *lax1* background, plants show strong phenotype. In the heterozygous *nsp1-D* and homozygous *nsp1-D* background, heterozygous *lax1* will enhance the morphological defects.

- In situ-hybridization experiments demonstrated normal LAX-1 expression in the bHLH triple mutant, qRT may have indicated slightly enhanced LAX-1 expression I the triple bHLH line (statistical analysis missing).
- Expression level of bHLH067,068 and 069 is not affected by the lax-1 mutation.

Response:

We have done the statistical analysis. In S5GFig, qRT-PCR result indicted that the expression of *OsbHLH067*, *OsbHLH068*, and *OsbHLH069* was affected in *lax1* plants.

- In yeast two-hybrid assays truncated versions of bHLH067/8/9 physically interacted with a truncated form of LAX1. The same interactions were found when only the bHLH domains of the respective genes were used.

Response:

Thanks for your comments.

We added another bHLH transcription factor OsbHLH003 as a negative control (Fig 7B).

- In bimolecular fluorescence complementation (BiFC) assays the interactions of the bHLH-fragments could be confirmed.

Response:

We have taken your suggestion and confirmed the interaction by in vitro pull-down assays (Fig 7C)

- RNASeq experiments using material from wild-type and triple bHLH-mutants resulted in a high number of differentially expressed genes.
- Among the genes up- or down-regulated in the triple-mutant, several functional categories are enriched. One category was significantly enriched in the genes down-regulated (Protein processing in endoplasmic reticulum; Q value=9.77E-04), several categories were enriched in the genes up-regulated in the triple mutant.

Response:

Because OsbHLH067, OsbHLH068, and OsbHLH069 are typical transcription factor harboring transcriptional activation activities, we focused on the examination of these down-regulated genes in the triple mutant *Osbhlh067 Osbhlh068 Osbhlh069*.

It is interesting that up-regulated genes were significantly enriched only in “protein processing in endoplasmic reticulum”. We will examine their function in the future.

Do the experiments confirm statements of the authors?

1. A weak point of the manuscript is the fact, that there is no explanation for the contradicting branching defects: Over-expression of bHLH069 leads to reduced lateral meristem formation in the inflorescence. This indicates a repressing function of bHLH069 in meristem branching. A combination of loss-of-function alleles for bHLH067/068/069 leads to reduced inflorescence branching. This indicates an activating function of bHLH069 in meristem branching.

This question is not addressed in the paper.

Response:

Thanks for your comments.

LAX1 is a key factor in determining the formation of AM in rice. *LAX1* expression must be finely regulated for the normal AM formation. Mutation in *LAX1* severely suppresses the initiation of lateral spikelets and affects both vegetative and reproductive branching. Over expression of *LAX1* also causes reduced branching (Komatsu K et al. *LAX* and *SPA*: major regulators of shoot branching in rice. Proc Natl Acad Sci U S A. 2003; 100:11765–70). In our study, the interaction between *OsbHLH067/OsbHLH068/OsbHLH069* and *LAX1* for AM formation was investigated. Like the LAX1, we think the activity of OsbHLH067/OsbHLH068/OsbHLH069 also should be fine regulated. We have discussed it in the last paragraph in the revised version.

2. The authors need to give a functional explanation or at least a hypothesis for the results of the different experiments testing the interaction of bHLH067/68/69 and LAX1: Double mutant analysis shows mutual enhancement of mutant phenotypes, which is most easily explained, when LAX1 and the three bHLH genes act in independent pathways/in parallel. Y2H and BiFC show a physical interaction of partners – the partners act in a protein complex. Either both partners are needed to have an active complex or one of the partners inactivates the other when forming a complex. In the first case, removing one of the partners should have the same effect as removing both (no enhancement of phenotypic defects). In the second case, effects of mutations

should be antagonistically more branching in one of the mutants, less branching in the other). This question needs to be addressed.

Response:

Thanks for your comments.

We have discussed it in the last paragraph in the revised version. *OsbHLH067/068/069* preferentially accumulated in the developing meristem and their protein interacted with LAX1. Thus, we propose that OsbHLH067/068/069-LAX1 modules is essential to regulate AM development in rice. With the onset of AM development, boundary expressed *LAX1* is required to participate in plant hormone signal transduction to promote AM initiation; during the AM development stage, OsbHLH067/068/069 interacts with LAX1 and are involved mainly in metabolism pathways, such as starch and sucrose metabolism, which might provide the nutritional signaling to maintain AM development.

3. The authors try to clarify the function of bHLH067/68/69 by analyzing transcript accumulation in the triple bHLH mutant. They find functional categories of genes enriched in up- or down-regulated genes. Why did they select a category with a relatively poor Q value for further analysis and not the one with the highest Q-value?

Response:

In the revised version, we examined the expression levels of those selected genes sorted by Q value. The down-regulated genes were confirmed in the triple mutant *Osbhlh067 Osbhlh068 Osbhlh069* (Fig 8D and 8E). Interestingly, all the examined genes involved in sucrose metabolism were significantly down-regulated in *lax1* (Fig 8F). Thus, we speculate OsbHLH067/068/069-LAX1 modules might be involved in starch and sucrose metabolism process for modulating AM development.

As the authors seem to be interested to define direct targets of bHLH067/68/69, they should try to clarify, if these bHLH proteins are transcription activators or repressors. Based on the description of the Y2H experiments they are acting as transcription activators. This would indicate that the category “Protein processing in endoplasmic

reticulum”, down-regulated in the triple-mutant would be the best candidate for further analysis. The authors need to address this question.

Response:

It is a nice suggestion. We will further examine that in the future.

Because OsbHLH067, OsbHLH068, and OsbHLH069 are typical transcription factor harboring transcriptional activation activities, we focused on the examination of these down-regulated genes in the triple mutant *Osbhlh067 Osbhlh068 Osbhlh069*.

It is interesting that up-regulated genes were significantly enriched only in “protein processing in endoplasmic reticulum”.

Minor concerns:

The authors should try to explain the differences/contradictions between qRT-PCR experiments and RNA in-situ hybridization.

Response:

Quantitative RT-PCR results showed that *OsbHLH067*, *OsbHLH068*, and *OsbHLH069* are constitutively expressed in various organs, including root, culm, leaf, leaf sheath, and young panicles of less than 5 mm. To examine their expression on young panicle tissue levels, *in situ* hybridization suggest that *OsbHLH067/068/069* is preferentially expressed in inflorescence AM (Fig 4).

The authors need a different negative control for Y2H and biFC. bHLH proteins have a tendency to form dimers. Binding of proteins is a chemical process. Using unnatural high concentrations of proteins that would not interact in natural concentrations may indicate interactions that are not relevant under

As bHLH-genes are numerous, it is necessary to prove that LAX binds better to bHLH067/68/69 than to other bHLH proteins not linked to branching. This would mean, that the negative control for Y2H and biFC would not be an empty vector control.

Response:

Thanks for your comments. We used another bHLH transcription factor OsbHLH003 as a negative control (Fig 7B).

The explanations, why phenotypes of genes with sequence similarity to bHLH067/68/69 in Arabidopsis thaliana have not been linked to similar phenotypes (branching vs. salt stress response and flowering transition; page 16) are unclear.

Response:

The plant bHLH family, a large family of transcription factors, can be divided into 32 subfamilies. Thus, the bHLH genes in the same subfamily potentially having redundant but distinct functions in rice and Arabidopsis, presumably due to the evolutionary functional divergence of homolog-encoded proteins.

RNA-Seq experiments: Information is insufficient: Materials and Methods does not describe the analysis in sufficient detail to judge the results.

Response:

We have added the methods pull-down assay, RT-PCR, and histological section in the Material and methods.

Why are only 6740 genes included in the DeSeq-analysis? What filters have been used to obtain the number 6740 genes?

Response:

In rice genome, there are only 6740 genes have been annotated by KEGG pathway.

Page 30 Accession numbers: These are gene names, but not accession numbers. I tried to download the corresponding sequences and found this difficult. As gene names change, accession numbers must be used.

Response:

Thanks for your comments. We have provided the website. Sequence data in this study can be found in the Rice Genome Annotation Project (http://rice.uga.edu/).

References to supplemental tables S2 and S3 need to be switched in lines 290 and 292.

Response:

Yes, we have checked that.

Description of qRT is missing in Materials and Methods.

Response:

Yes, we have added the description of qRT in Materials and Methods.

In addition it has been demonstrated, that using a single reference gene can give wrong results, especially when comparing expression levels between different tissues.

Response:

It is a nice comment. The house-keeping gene used in this study seems showed the same expression levels among different tissues.

Reviewer #2:

Aerial plant architecture is largely depended on the axillary meristem development. In monocot plant rice, axillary meristems give rise to tiller formation during vegetative stage. After transition to reproductive stage, axillary meristems development determines panicle branching and spikelet formation. Tiller number, panicle size and spikelet are important agronomic traits and deeply influence yield. In this manuscript, Xu et al., identified a dominant sparse spikelet mutant from a T-DNA insertion library. The mutants were compromised in panicle branching development and exhibited severe defects in spikelet formation in a dosage-dependent manner. Further genetics evidence suggests that sparse spikelet defects were due to overexpression of *OsbHLH69* gene. They found that OsbHLH69 and its closely homologs OsbHLH67 and OsbHLH68 were expressed in almost all reproductive meristems. In addition, three genes regulate rice panicle development redundantly and triple mutant displayed severe defects, especially small panicles with less branching and spikelet. They also showed that OsbHLH69 can physically interacts with LAX1, the major regulator of axillary meristem formation in rice, and OsbHLH69 may have a synergistic effect with LAX1 on rice axillary meristem formation. Through RNA-seq analysis, they claimed OsbHLH67/68/69 may regulate rice panicle development through metabolism pathways.

Rice bHLH transcription factor family has many members and they usually work redundantly. The manuscript provides some interesting data about functions of three bHLH transcription factor on rice panicle formation. However, some designs were not well established and some conclusions are confused. There are several explanations about the defects in *nsp1-D* and the triple mutant, for example, all axillary meristems are not well initiated or some types of axillary meristems could not specified, like spikelet meristem could not normally developed. I think it is very important that the authors should clearly define defects in *nsp1-D* and triple mutants.

1. In rice, after transition to reproductive stage, axillary meristem phase change has important roles in panicle structure. Identities of branching meristems and spikelet meristem are different. To better understand panicle architecture, it is crucial to check different axillary meristems in a spatial order. As shown in figure 1H, I and L, M,

branching meristems seems normally formed, but in *nsp1-D* mutants, branching meristem could not develop into spikelet meristem. I would suggest the authors check the development process in more details. For example, check the SAM morphology with more development stages, better includes 1. vegetative, 2. inflorescence meristem, 3. young inflorescence meristem at the early primary branch initiation stage, 4. young inflorescence meristem at the late primary branch initiation stage and 5. immature inflorescence meristem at the spikelet meristem initiation stage.

Response:

Thanks for nice comments.

We have done the histological section of *nsp1-D* young panicles (Fig 2E-H) in the revised version. Combined with the SEM results, we observed that the primary branch meristems, second branch meristems and spikelet meristems are compromised in various degree.

2. In order to clarify the development defects in *nsp1-D* and triple mutants, besides using *OSH1* as a marker gene, I would suggest to use more markers to clearly define axillary meristem identities, for example, TAWAWA1 as branch meristem maker, and FZP and/or LHS1(OsMADS1) as spikelet meristem specific marker.

Response:

It is a good suggestion.

In this manuscript, we focus on the gene identification of *NSP1*. We found that *OsbHLH067, OsbHLH068,* and *OsbHLH069* function redundantly to regulate AM formation. In further study, we will take you suggestion to delve more deeply into the function of *OsbHLH069* by using the spikelet meristem specific marker *TAWAWA1*, *FZP1*, and *LHS1*.

3. *nsp1-D* mutants exhibited more defects in reproductive stage, while triple mutants presented defects in both vegetative and reproductive stage. It will be interesting to check transcriptome profiling also in *nsp1-D*. Importantly, I would suggest authors pay attention to different MADS-box family members, as some MADS-box gene inhibit

spikelet meristem formation (like SVP related genes), while some trigger the process (like SEP3, AG related genes). MADS-box gene expression profile could give more evidence to explain defects of *nsp1-D* and triple mutant.

Response:

It is a good suggestion.

We will further check the transcriptome profiling in *nsp1-D* in the following study. As you say, examination of MADS-box gene expression profile could provide more evidence to explain defects of *nsp1-D* and triple mutant.

Minor:

1. Line 60- 61, As known in Arabidopsis, STM keeps the boundary cells in a competent stage which is required for AM initiation. I think it is not necessary to put “P4” here, and better change “generated” to “initiated”.

Response:

Yes, we have corrected it.

2. Line 74-75, MOC1 is also an important regulator for reproductive AM formation.

Response:

We have modified it. MOC1 is a transcriptional regulator of the GRAS family and regulates the vegetative and reproductive AM formation in rice.

3. For the interaction between LAX1 and OsbHLH69, it is better to have an in-vitro pull down here as Y2H used the truncated protein.

Response:

Yes, we have taken your suggestion and done the in-vitro pull down assay. Pull down assays confirmed the interaction between OsbHLH69/067/068 and LAX1 (Fig 7D and 7E).
