# Supplementary material for: OsbHLH067, OsbHLH068, and OsbHLH069 redundantly regulate inflorescence axillary meristem formation in rice
Source: PLoS Genet. 2023 Apr 13;19(4):e1010698. doi: 10.1371/journal.pgen.1010698 (PMC10128955; doi:10.1371/journal.pgen.1010698)
Supplement: Attachment — Submitted filename: Response.pdf [file pgen.1010698.s015.pdf]

Dear Editor and Reviewers,

Thanks for all the constructive criticisms and comments. We have addressed them in this revised version. Below is a point-by-point response to all comments.

Editor synopsis:

1. Both reviewers found that the majority of their comments were unaddressed, which make both of them unsatisfied with the revision. There are two types of concerns. The first group of concerns is about the principles of axillary meristem development. As mentioned by Reviewer 2, vegetative and reproductive stage axillary meristem are regulated by distinct groups of genes. Also, branch meristems and spikelet meristems are different. The second group of concerns is about clarification of the OsbHLH genes function. Reviewer 3 suggests clarifying the difference between overexpressor and mutant phenotypes, and using qRT-PCR analysis to verify the expression of the downregulated genes in nsp1-D and other mutants. I would like to ask the authors to at least address the above mentioned points.

   Considering the long growth cycle of rice and difficulty of generation multiple mutants and harvesting inflorescence tissue, we apologize for not doing all the experiment suggested by two reviewers. We supplemented the experiment directly related this manuscript. Some unfinished experiments have been explained in the response.

   We have been more precise with definition of axillary meristem (AM) development. The AM specifically referring to the reproductive development stage has been defined as 'inflorescence AM'. We also notice that branch meristems and spikelet meristems are different. We have modified the text to be more specific.

   In our study, *OsbHLH067*, *OsbHLH068*, *OsbHLH069* and *OsbHLH070* belong to the subfamily F of the bHLH transcription factor in rice, although *OsbHLH070* does contain an atypical bHLH domain with no DNA binding ability. Because we

have not obtained the *Osbhlh070* mutant, the role of *OsbHLH070* in inflorescence AM development cannot be ruled out. This has been discussed in lines 335-340

2. A minor point: Arabidopsis auxin influx carrier LAX1 mentioned in the Introduction is not relevant to the paper, and more importantly confusing with rice LAX1 that regulates spike branching.
   Thanks for your suggestion. We have modified that (Line 92).

Reviewer #2: Xu et al., addressed some points in the revised version, but I think there are still some points should be discussed further.

1. Vegetative axillary meristems (AMs) and reproductive AMs development are quite different in rice. Both bHLH69 over-expression lines and bHLH67/68/69 triple mutants exhibited branching defects, while triple mutants are compromised in vegetative and reproductive stages. I think it is important to discuss in more details. And I suggest the authors to clearly clarify branching defects caused by AM initiation or outgrowth, eg, discussion part from line 406, it is rather confusing if AM initiation or outgrowth are not discussed separately.
   We agree with this criticism. We have now reorganized this part of the discussion from line 426 to 441. We focused on the functional differences between *LAX1* and *OsbHLH067/068/069* in the initiation and maintenance of inflorescence lateral meristem. We have now added qRT-PCR results of some auxin-related genes expression (S6 Fig). We speculated that *LAX1* is likely to be involved in auxin pathway to the initiation of inflorescence AM development, *OsbHLH067/068/069* might be required to maintain the inflorescence AM development. Based on the expression pattern of *OsbHLH067/068/069*, we have discussed that *OsbHLH067/068/069* may have multiple functions in regulating reproductive branching (Line 439-446).

2. In rice, identities of branching meristems and spikelet meristem are different. As shown here that branching meristems seems normally formed in nsp1-D mutants,

but could not develop into spikelet meristem. The determinacy balance between inflorescence meristem and spikelet meristem are very important for final panicle structure. It is essential to discuss the potential role of newly identified bHLH69 and its homologs in regulating the balance between inflorescence termination and spikelet meristem formation.

We agree with this criticism. Rice panicle development is set to undergo an interned process, that is IM initiates indeterminate branch meristems (BMs), which in turn produce a series of spikelet meristems. The specific functions of OsbHLH69 in spikelet meristem and AM meristem development depend on the examination of some marker gene expression profiles, which is just as the third reviewer suggested. We are currently examining some spikelet meristem marker gene expression profiles in nsp1-D mutants and the triple mutant, but this work is still ongoing and out scope here. We have discussed that in this version (Lines 444-446).

3. bHLH67/68/69 are widely expressed in almost whole meristem, and LAX1 is mainly expressed in the boundary region. Even though LAX1 protein could move, but it is still difficult to conclude real biological function of the interactions between LAX1 and bHLH69 related proteins. I think the interaction part somehow leads to more complicate to understand function of bHLH69 and its related homologs.

   Thanks for your suggestion, we do agree that. Our speculation is only based on the analysis of RNA-Seq data and RT-PCR. The specific molecular pathway of OsbHLH69 needs further experiments. Unfortunately, these studies are incomplete and far from being ready for publication.

Reviewer #3: Overall, this study will be a beneficial supplement to the field. I can see that the language has been substantially improved and the authors have provided more details in Materials and Methods section.

However, the underlying mechanism of the OsbHLH 067/068/069-OsLAX1 module is still lacking. In addition, the concerns raised by me and other reviewers were not fully addressed. For nearly all the major concerns, the authors did not perform additional experiments to answer those questions, but threw them off very easily and promised casually to solve them in their future study. As I mentioned in last review, the authors need to reconcile the apparent discrepancy before the work can be accepted.

Major concerns

1. L201 The authors claimed that OsbHLH070 does not contain a bHLH domain, which is different from their response to reviews and the alignment shown in Fig S1B. Even given the bHLH domain in OsbHLH070 is longer than those in the other three paralogues, the functions of OsbHLH070 are not necessarily different from the other three. The authors should not have precluded this gene in their functional characterization.

   Yes, we agree that *OsbHLH070* should not be precluded in their functional characterization.

   bHLH TFs with less than 5 basic amino acids in the basic region are incapable of DNA binding (Massari and Murre 2000). *OsbHLH070* does contain this atypical bHLH domain and was regard as with no DNA binding ability. Thus, we gave priority to generate OsbHLH067, OsbHLH068, OsbHLH069 mutants by CRISPR/Cas9. Because we have not obtained the *Osbhlh070* mutant, the role of *OsbHLH070* in inflorescence AM development cannot be ruled out. We have discussed that in lines 328-333.

2. The authors should perform in situ hybridization for OsbHLH067/068/069 in lax1 mutant to exclude the transcriptional regulation of LAX1 on OsbHLH067/068/069.

We agree with this criticism. Although our qRT-PCR analysis showed that the expression of *OsbHLH067*, *OsbHLH068*, and *OsbHLH069* was not affected in *lax1* plants (S5H Fig), in situ hybridization would be better to answer the transcriptional regulation of LAX1 on *OsbHLH067/068/069*. Due to the long growth cycle of rice and difficulty of harvesting inflorescence tissue, we apologize for not including this experiment in this manuscript.

3. The authors should perform in situ hybridization for representative genes (such as those required for AM initiation and sucrose metabolism) identified from RNA-seq analysis in the background of Osbhlh triple mutant and lax1 mutant, especially because the authors claimed them to be downstream genes of OsbHLH067/068/069 and LAX1. qRT-PCR is not sufficient.
Indeed, this is the case. It would be better to do in situ hybridization. It is more difficult to do in situ hybridization experiment in rice than that in Arabidopsis, mainly due to the long growth cycle of rice and the difficulty of harvesting young panicle (0-2 mm). In rice molecular biology research, the differentially expressed genes produced by RNA-Seq are usually verified by qRT-PCR and rarely by in situ hybridization, except for the direct target genes.

4. The authors should explain why both the nsp-1D, representing an overexpression line of OsbHLH069, and the osbhlh067 068 069 triple mutant show defective AM initiation. The authors should focus on the differences rather than the common behavior between the two types of mutants. To this end, they should focus on the indeterminacy of distinct types of meristems in different mutants. Checking the expression of more meristem markers by in situ hybridization such as TAWAWA1, FZP and LHS1 may help distinguish the subtle differences between them. Furthermore, similar to the phenotypic analysis for nsp-1D, at least SEM or histological observations against loss-of-function mutants should be included to address this.

Many thanks for your helpful suggestions, which will guide our follow-up research. Of course, using marker genes of different meristems doing in situ hybridization will precisely describe the inflorescence defects between the *nsp-1D* and the triple mutant. Based on our existing data, we only discussed why overexpression of OsbHLH069 and inhibition of its homologous family genes lead to defects in AM development (Lines 431-441). Like rice *LAX1*, these results indicated that the comprehensive genetic networks associated with inflorescence architecture must be precisely regulated.

5. Considering that the expression region of OsbHLH067/068/069 mRNA is much wider than that of LAX1, the loss of function mutants of the bHLH genes would lead to defects other than that seen in lax1 mutant. The simple OsbHLH067/068/069-LAX1 module is not sufficient. The authors should create translational reporters, to show that in planta the protein products coexist in leaf axils or rising meristems.

   We agree with this criticism. We have done the experiments Y2H, BiFC, and pull-down assays to confirm the interaction of OsbHLH067/068/069 and LAX1. If their interaction could be verified in planta, OsbHLH067/068/069-LAX1 module will be better convince.

6. The genetic interactions between OsbHLH067/068/069 and OsLAX1 should be characterized by generating quadruple mutant containing Osbhlh067 068 069 and oslax1 loss of function mutations and analyzing the corresponding phentoypes. This is also the experiment suggested by Reviewer 1.

   Even the homozygous of *lax1* cannot generated seeds, we have attempted to generate the quadruple mutant containing *Osbhlh067 068 069* and *lax1* by crossing their heterozygous plants. However, *OsbHLH069* (LOC_Os01g57580) and *LAX1* (LOC_Os01g61480) are physically located on chromosome 1, it is hard to obtain their double mutant through chromosome recombination. Of course, generating quadruple mutant would be better analyzing the corresponding phenotypes.

7. To conclude that the downregulated genes are actually downstream of OsbHLH067/068/069, it is necessary to perform at least qRT-PCR analysis is necessary to examine the expression of the downregulated genes in nsp1-D and/or the generated bHLH067/068/069 overexpression lines.

   Actually, we have selected some downstream genes to detect their expression in nsp1-D, and found that most of them were also down-regulated in nsp1-D. Due to the interaction between bHLH067/068/069 and LAX1, overexpression of bHLH069 may affect the regulatory activity of LAX1. In addition, bHLH067/068/069 and LAX1 both jointly or independently regulate the molecular network of AM formation, which is the evidence that their mutants and overexpressed plants showed the AM developmental defects. We apologize that we cannot obtain more data to answer this point.

Minor concerns

1. A number of highlighted regions are identical to last version or not pinpointing at all. The authors should pinpoint what has been changed and to which review is the revision relevant.

   We provided a mark-up copy containing the last two revisions in this version. This time, the revision was tracked with 'Track Changes' model. The specific line containing revision was also point out in the response.

2. The third paragraph in the Introduction (L86-98), which focuses on auxin biosynthesis, transport and signaling, does not seem to be relevant to the study. None of the genes or pathways were involved in the phenotypes.

   Auxin biosynthesis, transport, and signaling have been demonstrated to be required for inflorescence AM formation and lateral organ initiation. Considering that rice *LAX1* is the ortholog of *BA1*, we hypothesized that *LAX1* acts a conserved mechanism in boundary domains for AM formation by the auxin pathway. We have

now added qRT-PCR results of some auxin-related genes expression in this version (Lines 419-425). qRT-PCR analysis suggested some auxin-related genes were consistently suppressed in *lax1* plants (S6A Fig). In contrast, most of them have normal expression levels in the *Osbhlh067 Osbhlh068 Osbhlh069* triple mutant (S6B Fig).

3. L89 PID is different from PIN1 and AUX/LAX and not an auxin carrier, as pointed out in last review.

   Thanks for this observation. We have modified the text to be more specific (Line 90).

4. L337 obviously -> obvious, as pointed out in last review.

   Thanks for this observation. We have modified that (Line 342).

5. L338 observation ->observations

   Thanks for this observation. We have modified that (Line 343).

6. L529 Gutathione -> Glutathione

   Thanks for this observation. We have modified that (Line 550).
