# Supplementary material for: OsbHLH067, OsbHLH068, and OsbHLH069 redundantly regulate inflorescence axillary meristem formation in rice
Source: PLoS Genet. 2023 Apr 13;19(4):e1010698. doi: 10.1371/journal.pgen.1010698 (PMC10128955; doi:10.1371/journal.pgen.1010698)
Supplement: Attachment — Submitted filename: Comment on Xu et al., .docx [file pgen.1010698.s012.docx]

Aerial plant architecture is largely depended on the axillary meristem development. In monocot plant rice, axillary meristems give rise to tiller formation during vegetative stage. After transition to reproductive stage, axillary meristems development determines panicle branching and spikelet formation. Tiller number, panicle size and spikelet are important agronomic traits and deeply influence yield. In this manuscript, Xu et al., identified a dominant sparse spikelet mutant from a T-DNA insertion library. The mutants were compromised in panicle branching development and exhibited severe defects in spikelet formation in a dosage-dependent manner. Further genetics evidence suggests that sparse spikelet defects were due to overexpression of *OsbHLH69* gene. They found that OsbHLH69 and its closely homologs OsbHLH67 and OsbHLH68 were expressed in almost all reproductive meristems. In addition, three genes regulate rice panicle development redundantly and triple mutant displayed severe defects, especially small panicles with less branching and spikelet. They also showed that OsbHLH69 can physically interacts with LAX1, the major regulator of axillary meristem formation in rice, and OsbHLH69 may have a synergistic effect with LAX1 on rice axillary meristem formation. Through RNA-seq analysis, they claimed OsbHLH67/68/69 may regulate rice panicle development through metabolism pathways.

Rice bHLH transcription factor family has many members and they usually work redundantly. The manuscript provides some interesting data about functions of three bHLH transcription factor on rice panicle formation. However, some designs were not well established and some conclusions are confused. There are several explanations about the defects in *nsp1-D* and the triple mutant, for example, all axillary meristems are not well initiated or some types of axillary meristems could not specified, like spikelet meristem could not normally developed. I think it is very important that the authors should clearly define defects in *nsp1-D* and triple mutants.

1. In rice, after transition to reproductive stage, axillary meristem phase change has important roles in panicle structure. Identities of branching meristems and spikelet meristem are different. To better understand panicle architecture, it is crucial to check different axillary meristems in a spatial order. As shown in figure 1H, I and L,M, branching meristems seems normally formed, but in *nsp1-D* mutants, branching meristem could not develop into spikelet meristem. I would suggest the authors check the development process in more details. For example, check the SAM morphology with more development stages, better includes 1. vegetative, 2. inflorescence meristem, 3. young inflorescence meristem at the early primary branch initiation stage, 4. young inflorescence meristem at the late primary branch initiation stage and 5. immature inflorescence meristem at the spikelet meristem initiation stage.

2. In order to clarify the development defects in *nsp1-D* and triple mutants, besides using *OSH1* as a marker gene, I would suggest to use more markers to clearly define axillary meristem identities, for example, TAWAWA1 as branch meristem maker, and FZP and/or LHS1(OsMADS1) as spikelet meristem specific marker.

3. *nsp1-D* mutants exhibited more defects in reproductive stage, while triple mutants presented defects in both vegetative and reproductive stage. It will be interesting to check transcriptome profiling also in *nsp1-D*. Importantly, I would suggest authors pay attention to different MADS-box family members, as some MADS-box gene inhibit spikelet meristem formation(like SVP related genes), while some trigger the process(like SEP3, AG related genes). MADS-box gene expression profile could give more evidences to explain defects of *nsp1-D* and triple mutant.

Minor:

1. Line 60- 61, As known in Arabidopsis, STM keeps the boundary cells in a competent stage which is required for AM initiation. I think it is not necessary to put “P4” here, and better change “generated” to “initiated”.

2. Line 74-75, MOC1 is also an important regulator for reproductive AM formation.

3. For the interaction between LAX1 and OdbHLH69, it is better to have an in-vitro pull down here as Y2H used the truncated protein.
