# Supplementary material for: OsbHLH067, OsbHLH068, and OsbHLH069 redundantly regulate inflorescence axillary meristem formation in rice
Source: PLoS Genet. 2023 Apr 13;19(4):e1010698. doi: 10.1371/journal.pgen.1010698 (PMC10128955; doi:10.1371/journal.pgen.1010698)
Supplement: Attachment — Submitted filename: Response to the reviewers 2.docx [file pgen.1010698.s014.docx]

Reviewer #2: The revised version by Xu et al., addressed some points, but I think there are still some concerns are not well addressed.

1. As shown in the manuscript, OsbHLH69/68/67 are important for rice panicle development, and both over-expression and loss of function mutants are compromised in panicle development. During rice panicle development, primary and secondary branching meristems still have the meristematic ability, but spikelet meristems are somehow developed into a terminate status. So it is very crucial to clearly clarify the defects of these mutants. As I suggested last time, OSH1 is not a good marker here, it is better to use some more specific marker, TAWAWA1 as branch meristem maker, and FZP and/or LHS1(OsMADS1) as spikelet meristem specific marker. And I would also suggest the authors discuss potential roles of OsbHLH69/68/67 in meristem determinacy or indeterminacy regulation.

Response:

Thanks for your nice suggestion.

Our investigation focuses on the identification of OsbHLH067, OsbHLH068, and OsbHLH069 gene in rice. We used OSH1 as a marker gene for meristematic cells. We found that OsbHLH067/068/069 act redundantly to regulate the formation of inflorescence axillary meristems. In future studies, we will take you suggestion to delve more deeply into the function of OsbHLH067/068/069 by using the spikelet meristem specific markers TAWAWA1, FZP1, and LHS1.

2. In figure 8, selected AM formation genes are not enough, and their functions are quite different. As I suggested, it is better to include nsp1-D mutant inside and check some key transition MADS-box genes.

Response:

Thanks for your nice suggestion.

Because we did not collect the young panicle tissues from *nsp1-D* mutant and WT plants to do the comparative RNA-seq, we will take your suggestion to check the transcriptome profiling in *nsp1-D* compared with WT in our next examination. As you suggested, examination of MADS-box gene expression profile could provide more evidence to explain defects of *nsp1-D* and triple mutant.

Minor concerns

1. Line 31, I would suggest to use a weaker statement, it is not convinced that the phenotypes of lax1 and nsp1-D are "similar".

Response:

Yes, we have corrected it.

2. For BiFC part, empty nCFP or cCFP are not suitable negative controls.

Response:

Thanks for your nice suggestion. We have realized that and will pay attention in the BiFC experiment. In this manuscript, we confirmed the protein interaction by *in vitro* pull-down assays.

Reviewer #3: The research article by Xu et al. described the isolation and cloning of a T-DNA insertion mutant with sparse-panicle phenotype. Besides identifying the causal gene locus, they characterized the mutant phenotype by morphological description and ISH and confirmed through molecular genetic approaches that the sparse-panicle phenotype was indeed induced by overexpression of a gene encoding a bHLH transcription factor. They further profiled the spatio-temporal expression patterns of this bHLH069 gene and its close paralogs bHLH067 and bHLH068, which likely function redundantly with bHLH069 in promoting AM initiation. They also found that the bhlh067 bhlh068 bhlh069 triple mutant resembles Oslax1 mutant in AM initiation potential, suggesting that these bHLH TFs might function in the same pathway with OsLAX1. They performed a number of in vitro assays to confirm that OsbHLH 067/068/069 interact with LAX1. They carried out high-throughput transcriptome profiling and found some potential targets of the common module composed of OsbHLH 067/068/069-OsLAX1.

Overall, this study is sound and interesting, not only to the field of crop sciences, but also to the field of plant development. It is definitely a beneficial complement to currently published studies. However, the underlying mechanism of the OsbHLH 067/068/069-OsLAX1 module is still lacking. There are still some major issues and quite some minor problems for the authors to address.

Major concerns

1. The authors need to provide a reasonable explanation for why both the nsp-1D, representing an overexpression line of OsbHLH069, and the osbhlh067 068 069 triple mutant show defective AM initiation. The authors should focus on the differences rather than the common behavior between the two types of mutants. Similar to the phenotypic analysis for nsp-1D, SEM or histological observations against loss-of-function mutants should be included to address this.

Response:

Thanks for your nice suggestion.

We have discussed the OsbHLH067/068/069 functions in the end of the discussion part in this revised version. Multiple investigations have demonstrated that bud outgrowth might be mediated by specific nutritional and hormonal signaling pathways. Our investigation suggests that both *OsbHLH069*-overexpressing plants and the *Osbhlh067 Osbhlh068 Osbhlh069* triple mutant showed defects in reproductive AM development, resulting in reduced branches and spikelets. Considering that OsbHLH067/068/069 physically interact with LAX1, we speculate that OsbHLH067/068/069 may affect LAX1 activity to fine-tune panicle AM formation. With the onset of AM development, the boundary expressed LAX1 is required for plant hormone signal transduction to promote AM initiation; at the AM development stage, OsbHLH067/068/069 interact with LAX1 and participate in hormone signal and metabolism pathways, fine-tuning the hormone and nutritional signaling to maintain AM development. In addition, both OsbHLH067/068/069 and LAX1 are bHLH transcription factors, and thus their self-regulation of molecular networks in meristem development requires further examination.

2. Is it possible that the triple mutant and the oslax1 mutant are defective in AM initiation mainly due to nutritional defects, which cause pleiotropic phenotypes including AM initiation defects, while in the overexpression lines the defect is caused by disequilibrium in OsbHLH-OxLAX1 complex?

Response:

Thanks for your nice suggestion. We will take you suggestion to examine that in the next investigation.

3. From the phylogenetic tree in figure S1, it can be inferred that OsbHLH068 is more similar to OsbHLH070, as compared to OsbHLH067 and OsbHLH069. It seems the authors had pre-excluded OsbHLH070 in the AM-regulating module for some reason, but failed to provid the reason in the current version. The authors need to fill this gap to show why and how they pre-excluded OsbHLH070 in their analysis. To this end, the in situ hybridization analysis against OsbHLH070 may be required.

Response:

Protein sequence alignment analysis revealed that the amino acids in the basic region of the bHLH domain of OsbHLH070 were longer than those in OsbHLH067, OsbHLH068, and OsbHLH069 (S1 Fig). In addition, it was predicted that OsbHLH070 has no ability to bind downstream DNA, while OsbHLH067, OsbHLH068 and OsbHLH069 have the ability to bind downstream genes [31]. Therefore, we speculate that OsbHLH067, OsbHLH068 and OsbHLH069 are more likely to participate in the same metabolic regulatory pathway. In subsequent studies, the OsbHLH070 gene can be cloned to further study its relationship with OsbHLH067, OsbHLH068 and OsbHLH069.

4. Considering that the expression region of OsbHLH067/068/069 mRNA is much wider than that of LAX1, the loss of function mutants of the bHLH genes would lead to defects other than that seen in lax1 mutant. The authors need to reconcile the discrepancy. To answer this question, it may be necessary to create translational reporters, i.e., fused proteins of OsbHLH/OsLAX1 and fluorescent protein tags, to show that the protein products coexist in leaf axils or rising meristems.

Response:

Thanks for your nice suggestion. We will take you suggestion to examine that in the next investigation.

5. The genetic interactions between OsbHLH067/068/069 and OsLAX1 should be characterized by generating the quadruple mutant containing Osbhlh067 068 069 and oslax1 loss of function mutations and analyzing the corresponding phenotypes. This is also the experiment suggested by Reviewer 1.

Response:

The *Osbhlh067 Osbhlh068 Osbhlh069* triple mutant not only showed reduction of spikelets and branches, but also decreases in seed fertility without seeds. In addition, the seed setting rate of *lax1* mutant was also relatively low. It is difficult to obtain their quadruple mutants. In this study, we created a double mutant of *nsp1-D/+* and *lax1*, and it showed enhancement of phenotype, suggesting that they have a synergistic effect.

6. To conclude that the downregulated genes are actually downstream of OsbHLH067/068/069, it is necessary to perform transcriptome analysis also in nsp1-D, or at least qRT-PCR analysis is necessary to examine the expression of the downregulated genes in nsp1-D and/or the generated bHLH067/068/069 overexpression lines.

Response:

We are sorry to collect the young panicle tissues from *nsp1-D* mutant and WT plants to do the comparative RNA-seq. RNA-seq analysis of the triple mutant showed that OsbHLH067/068/069 participate in multiple metabolic pathways. In the subsequent research, we will consider to conduct RNA-seq analysis on young panicles of wild type, *nsp1-D/+* and *nsp1-D*, and construct *OsbHLH069* label plants to conduct ChIP-seq to find their specific downstream regulatory genes.

Minor concerns

1. The language needs to be improved substantially.

Response:

Thanks for the suggestion. We have thoroughly polished the language with the assistance a native speaker.

2. Materials and methods need to be elaborated on. Please provide detailed information instead of referring readers to some other papers.

Response:

Thanks for the suggestion. The Materials and methods part has been provided with more detailed information, such as scanning electron microscopy (SEM) analysis，histology, *in vitro* pull-down assays, RNA extraction and quantitative RT-PCR.

3. The authors should use two or more reference genes in qRT analysis to avoid the bias induced by using only a single reference gene, the expression level might itself differ among tissues.

Response:

For some expression detections, *in situ* hybridization was also used for further judgement. As for the detection of expression profiles in different tissues, in addition to using ubiquitin as a reference gene for qRT-PCR detection, we also referred to the microarray data in <http://ricexpro.dna.affrc.go.jp>. The results showed that there was nearly no difference.

4. Line edits (should not limited to these listed ones)
L30: delete “which”
L31: LAX1 -> which, transcriptomic -> transcriptomic
L32: metabolism -> metabolic
L41: has -> have
L44: underling -> underlying
L89: homologues -> homologous
L90: mutation -> mutations； auxin pathways -> auxin signaling pathways
L92: OsPID is not an auxin transporter, but a kinase activating PIN1.
L96: delete “rice”, which appears twice in the same sentence； Factor -> factors
L137: performed -> exhibited the phenotype of weakly sparse inflorescences (same for L176)
L141: effects -> defects
L173: genotype -> insertion
L175: seriously -> severely
L232: statue -> stature
L253: transcription -> transcriptional
L259: less -> few
L289: performed -> perform
L301: factor -> factors
L314: functions -> function; interacts ->interact
L341: an obviously defects AM development -> an obvious defect in AM development
L350: In analyzing -> By analyzing
L357: delete “the”
L429: Isolation flanking -> isolation of the flanking

Response:

Thanks for the suggestions. We have corrected all of them and thoroughly polished the language with the assistance a native speaker.
